# Accuracy of identifying incident stroke cases from linked health care data in UK Biobank

**DOI:** 10.1212/WNL.0000000000009924

**Published:** 2020-08-11

**Authors:** Kristiina Rannikmäe, Kenneth Ngoh, Kathryn Bush, Rustam Al-Shahi Salman, Fergus Doubal, Robin Flaig, David E. Henshall, Aidan Hutchison, John Nolan, Scott Osborne, Neshika Samarasekera, Christian Schnier, Will Whiteley, Tim Wilkinson, Kirsty Wilson, Rebecca Woodfield, Qiuli Zhang, Naomi Allen, Cathie L.M. Sudlow

**Affiliations:** From the Centre for Medical Informatics, Usher Institute of Population Health Sciences and Informatics (K.R., K.B., R.F., A.H., J.N., C.S., T.W., K.W., Q.Z., C.L.M.S.), and Centre for Clinical Brain Sciences (R.A.-S.S., F.D., N.S., W.W., R.W.), University of Edinburgh; UK Biobank (K.R., K.B., R.F., A.H., J.N., C.S., T.W., K.W., R.W., Q.Z., N.A., C.L.M.S.), Stockport; University of Edinburgh Medical School (K.N., D.E.H., S.O.); and Nuffield Department of Population Health (N.A.), University of Oxford, UK.

## Abstract

**Objective:**

In UK Biobank (UKB), a large population-based prospective study, cases of many diseases are ascertained through linkage to routinely collected, coded national health datasets. We assessed the accuracy of these for identifying incident strokes.

**Methods:**

In a regional UKB subpopulation (n = 17,249), we identified all participants with ≥1 code signifying a first stroke after recruitment (incident stroke-coded cases) in linked hospital admission, primary care, or death record data. Stroke physicians reviewed their full electronic patient records (EPRs) and generated reference standard diagnoses. We evaluated the number and proportion of cases that were true-positives (i.e., positive predictive value [PPV]) for all codes combined and by code source and type.

**Results:**

Of 232 incident stroke-coded cases, 97% had EPR information available. Data sources were 30% hospital admission only, 39% primary care only, 28% hospital and primary care, and 3% death records only. While 42% of cases were coded as unspecified stroke type, review of EPRs enabled a pathologic type to be assigned in >99%. PPVs (95% confidence intervals) were 79% (73%–84%) for any stroke (89% for hospital admission codes, 80% for primary care codes) and 83% (74%–90%) for ischemic stroke. PPVs for small numbers of death record and hemorrhagic stroke codes were low but imprecise.

**Conclusions:**

Stroke and ischemic stroke cases in UKB can be ascertained through linked health datasets with sufficient accuracy for many research studies. Further work is needed to understand the accuracy of death record and hemorrhagic stroke codes and to develop scalable approaches for better identifying stroke types.

Stroke is the second commonest cause of death worldwide and a major global cause of disability.^1^ Very large prospective population-based studies are needed to improve our understanding of its risk factors and causal associations.^2^

UK Biobank (UKB) is a prospective population-based cohort study with extensive phenotypic and genotypic information on >500,000 participants from England, Scotland, and Wales (ukbiobank.ac.uk). It is an open-access resource, established to facilitate research into the determinants of a wide range of health outcomes, particularly those of relevance in middle and older age.^3^

A cost-effective way of following UKB participants for disease outcomes is via linkages to routinely collected, coded, national administrative health datasets. UKB receives regularly updated linkages to national hospital admission and death record data for all participants, and to primary care data for a large and increasing subset. However, appropriate use of these data in research studies requires understanding of their accuracy. In a systematic review of published studies, we found that stroke-specific diagnosis codes in hospital admission and death record data generally have good accuracy. However, the studies varied widely in their settings and methodology, with very limited data about the accuracy of primary care codes or the effect of combining different data sources.^4^

We therefore conducted a validation study to assess the accuracy of ascertainment of incident stroke cases in UKB via linked coded national administrative health datasets (including primary care data), compared with diagnoses assigned following adjudication by clinical experts with access to the full free-text electronic patient records (EPRs).

## Methods

### Study population

We conducted the study in a subpopulation of 17,249 UKB participants in the Lothian region of southeast Scotland, all of whom are linked to national administrative health datasets (including hospital, primary care, and death record codes). This region encompasses the city of Edinburgh, where one of UKB's recruitment centers was located. Within this subpopulation, we identified all those with at least one code in their linked health data that indicated a stroke diagnosis after their recruitment to UKB.

### Stroke definition

We defined stroke according to the WHO definition: “rapidly developing clinical signs of focal (or global) disturbance of cerebral function, lasting more than 24 hours or leading to death, with no apparent cause other than that of vascular origin.”^5^

### Code selection and sources

We selected and included relevant codes from hospital admission, primary care, and death records up to the end of September 2015, the date at which data were complete for all sources at the time of this study.

In the United Kingdom, hospital admissions and death records are coded by specialized medical coders, who use the International Classification of Diseases (ICD; currently version 10) coding system to assign a primary code to the main condition resulting in a particular admitted episode of care, and secondary codes to other conditions. Primary care data are coded by general practice clinical or administrative staff during clinical encounters or on receipt of information from elsewhere (e.g., hospital inpatient and specialist outpatient settings).

Primary care data in the United Kingdom currently use the Read coding system (version 2 in Scotland during the time period of relevance in this study).

We selected relevant stroke codes from the ICD-10 and Read version 2 coding systems, aiming to ascertain cases of stroke with a high positive predictive value (PPV) (i.e., to minimize the proportion of false-positives among the stroke cases), while aiming to ascertain as many as possible of the true cases (i.e., optimizing sensitivity). Our code selections were informed by the results of a systematic review,^4^ browsing of the Technology Reference Data Update Distribution Service (isd.hscic.gov.uk/trud3/user/guest/group/0/home), the Secure Anonymised Information Linkage databank cerebrovascular diseases dictionary,^6^ the Quality Outcomes Framework indicator sets for stroke (www.wales.nhs.uk/), and the CALIBER online data portal (www.caliberresearch.org/), as well as manual review of code lists by experts (table e-1, dryad.org/10.5061/dryad.w9ghx3fk0).

We only validated diagnoses for cases with their first incident stroke, defined as the first-ever-in-a-lifetime occurrence of a WHO-defined stroke according to codes from linked national administrative datasets. We excluded participants who self-reported a stroke at baseline recruitment or who had a stroke code in their linked health care data predating recruitment to UKB. This was because (1) incident disease cases (i.e., new-onset disease arising in those without a history of the relevant condition) are of most research interest in a prospective, population-based study recruiting mainly healthy volunteers and (2) a large proportion of stroke cases identified through codes or self-reported stroke status arising prior to recruitment (prevalent cases) predated the EPR system used for validation. We based our analyses on the earliest code(s) for each participant from any data source.

### Process of assigning an expert diagnosis

For each included participant with a stroke code, we extracted, anonymized, and created a single document (hereafter referred to as a “vignette”) from all available relevant medical information from the secondary care EPR system, including outpatient clinic letters, hospital discharge summaries, and formal radiologic investigation reports (figure e-1, dryad.org/10.5061/dryad.w9ghx3fk0). We considered information relevant if it was associated with a hospital admission leading to the code, or if it was within 2 months before or after the date of a relevant primary care code. A 2-month window was chosen to reflect local clinical practice during the time period of the study: patients in NHS Lothian with a suspected stroke or TIA not requiring hospital admission would have been referred to the neurovascular specialist outpatient clinic, where they would usually be seen within 1 week, in keeping with national guidelines (the National Institute for Health and Care Excellence guidance, www.nice.org.uk/guidance/cg68, and the Scottish Stroke Care Audit Standards, strokeaudit.scot.nhs.uk/Quality/Scottish_Stroke_Care_Standards.html).

Following this specialist clinic visit, correspondence to the patient's general practitioner would generally appear on the secondary care EPR system within days to a few weeks, providing the clinical information required to validate primary care codes.

We developed an adjudication form, which included a summary for adjudicators of criteria for the diagnosis of stroke, its pathologic types (ischemic stroke, intracerebral hemorrhage [ICH], subarachnoid hemorrhage [SAH]), and subtypes (appendix e-1, dryad.org/10.5061/dryad.w9ghx3fk0).

Six consultant stroke specialists (hereafter referred to as “adjudicators”) completed adjudication forms based on the information in the EPR-derived vignettes. They did not have access to review the brain imaging themselves, but formal scan reports were included in the vignettes. One adjudicator (K.R.) completed the forms for all participants and 5 other adjudicators (N.S., C.S., R.A.-S.S., F.D., W.W.) independently completed the forms (each for a subset) to allow assessment of interadjudicator agreement. All adjudicators were blinded to which specific codes had led to ascertainment of any case and to each other's diagnoses. The first adjudicator's diagnoses were used as the reference standard for the primary analyses in this study.

### Data analyses

#### Participants' characteristics

We summarized participants' sex and median age at recruitment and at the time the code was assigned.

#### Code source and type identification

We calculated the number and proportion of stroke-coded participants arising from each data source. We also stratified the codes into those specifying a stroke type (ischemic vs ICH vs SAH) vs those indicating an unspecified type of stroke, and calculated the numbers and proportions of these arising from each source. We compared these findings with the same analyses for the larger subset of all UKB participants with linkage to the relevant data sources in England, Scotland, and Wales.

#### Assessment of interadjudicator agreement

We used percent agreement and Cohen kappa (ĸ)^7^ to measure interadjudicator agreement, comparing one adjudicator's (K.R.) diagnoses with a second (one of N.S., C.S., R.A.-S.S., F.D., W.W.) for the following: stroke vs not; stroke/TIA vs not; and, in cases where both adjudicators agreed that the diagnosis was stroke, ischemic stroke vs ICH vs SAH vs uncertain stroke type.

#### Calculating code accuracy

The main measure of accuracy assessed in this study was PPV (the proportion of all stroke-coded cases ascertained that were true-positive cases). While we could not directly assess sensitivity, we were able to assess the effects on both PPV and the number of true-positive cases (a higher number of true-positives indicates higher sensitivity) of different code combination strategies for ascertaining cases. We categorized the stroke-coded cases as true-or false-positives based on adjudicators' diagnoses. We calculated the PPV and its 95% confidence interval (CI) using the Clopper Pearson exact method, considering both (1) only an adjudicator diagnosis of stroke a true-positive and (2) an adjudicator diagnosis of stroke or TIA a true-positive. To understand the contribution of each source of codes, we calculated PPVs for each code source separately (hospital admission vs primary care vs death record data) and for their combinations, and explored the effect (both on PPV and on true-positive case numbers) of including only codes in the primary position for hospital admission data.

We also calculated PPVs for ischemic and hemorrhagic stroke types, and explored the effect (both on PPV and on true-positive case numbers) of restricting hospital data to codes in the primary position and of treating all unspecified stroke type codes as ischemic stroke codes.

In further analyses, we assessed administrative vs overall code accuracy and the effect on resulting PPVs, as we hypothesized that this may explain some of the variability of the results among previous validation studies. Some validation studies are based on the assumption that if the diagnostic code reflects the diagnosis mentioned somewhere in the EPR (often, for example, the discharge summary for a particular hospital admission), then this is sufficient to confirm the accuracy of the code.^4^ We refer to this as the administrative accuracy of the code. Clinicians will, however, appreciate that assessing the accuracy of a diagnostic code is more nuanced. A patient's true diagnosis may emerge over time after encounters with several different clinicians with varying levels of expertise and with access to different amounts of relevant information; hence, the EPR can contain inconsistencies, which may require specialist clinical knowledge to detect and/or resolve. Hence a specialist physician's review of all relevant parts of the complete EPR will give a more accurate picture of the true diagnosis and so of the code accuracy, which we refer to as the overall accuracy. We assessed administrative accuracy by having a primary care physician in our team review the EPR-derived vignettes for mentions of stroke diagnoses. We assessed overall accuracy by having expert adjudicators (consultant level stroke specialists) review the EPR-derived vignettes and derive reference standard diagnoses based on all available information and their clinical interpretation of this information (see Process of assigning an expert diagnosis). We then assessed whether, and by how much, the administrative vs overall accuracy (PPV) differed for our selected stroke codes.

We performed all statistical analyses in R (r-project.org).

#### Further exploration of false-positive codes

We compared characteristics of participants with a true- vs a false-positive code and reviewed the vignettes of false-positive cases, considering their alternative diagnoses and assessing possible reasons for them being assigned a stroke code. Informed by these assessments, we evaluated alternative code combinations that we hypothesized may improve code accuracy.

#### Stroke type and subtype distributions

We further assessed the proportion of true-positive cases that could be assigned a pathologic stroke type (ischemic stroke, ICH, SAH) and more detailed subtype by the stroke specialist adjudicator, and their frequency distributions.

### Standard protocol approvals, registrations, and patient consents

All procedures performed in studies involving human participants were in accordance with the ethical standards of the institutional and/or national research committee and with the 1964 Helsinki declaration and its later amendments or comparable ethical standards. As part of the UKB recruitment process, informed consent was obtained from all individual participants included in the study.

### Data availability

Anonymized summary data based on this study not published within this article can be shared (subject to approval from UKB) on request from any qualified investigator unless limited by ethical or legal restrictions. Any bona fide researcher can access the UKB resource for research that aims to benefit the public's health (see ukbiobank.ac.uk).

## Results

### Study population

**Figure 1:**
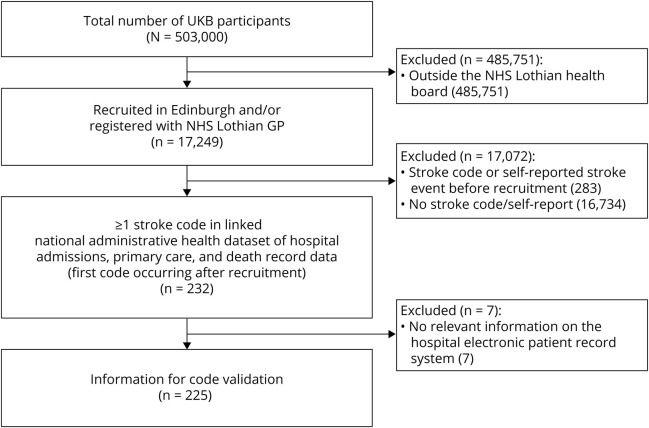
Selection of included UK Biobank (UKB) participants GP = general practitioner; NHS = National Health Service.

In the subpopulation of 17,249 participants, 232 had a relevant stroke code occurring after recruitment to UKB. Of these, 225 (97%) had available information in their EPR to create a vignette and were included in the study analyses (figure 1).

### Participants' characteristics

Of the 225 stroke-coded cases, 111 (49%) were female. Median age at recruitment to UKB was 63 years (range 41–70 years) and at time of the stroke code was 67 years (range 45–76 years) (table 1).

**Table 1:**
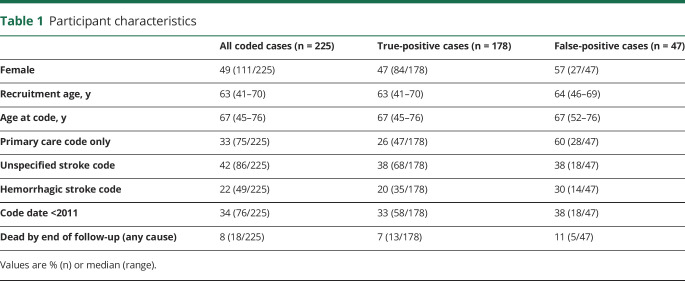
Participant characteristics

### Code sources and types

Of the 225 stroke-coded cases, 67 (30%) received a code from hospital admission data only, 87 (39%) from primary care data only, 64 (28%) from both hospital admission and primary care data, 6 (3%) from death records only, and one from both hospital admission data and death records (table 2 and figure e-2, dryad.org/10.5061/dryad.w9ghx3fk0). For the larger subset of all UKB participants with linkage to the relevant data sources, these proportions were similar for Scotland, but a higher proportion of participants in England and Wales had hospital-only codes (table 2).

**Table 2:**
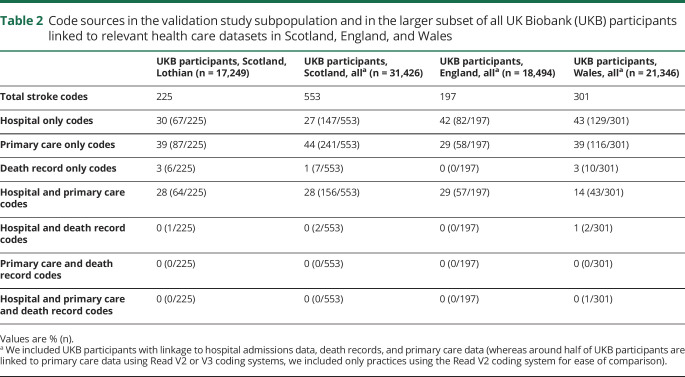
Code sources in the validation study subpopulation and in the larger subset of all UK Biobank (UKB) participants linked to relevant health care datasets in Scotland, England, and Wales

As regards stroke types, 131/225 (58%) of stroke-coded cases had a stroke type-specific code (ischemic, ICH, or SAH), while the remaining 94 (42%) had unspecified stroke codes. The proportion of cases with unspecified stroke type codes was higher among those ascertained from primary care (105/151 [70%]) than from hospital admission data (38/132 [29%]), and all death record codes were stroke-type specific (for these code-source level estimates, a case would be counted ≥1 if occurring in ≥1 source) (table 3 and figure e-2, dryad.org/10.5061/dryad.w9ghx3fk0). Among the larger subset of all UKB participants with relevant linked data, the proportion of cases with an unspecified stroke code was similar for Scotland, but lower for England and Wales (19% and 30%, respectively) (table 3).

**Table 3:**
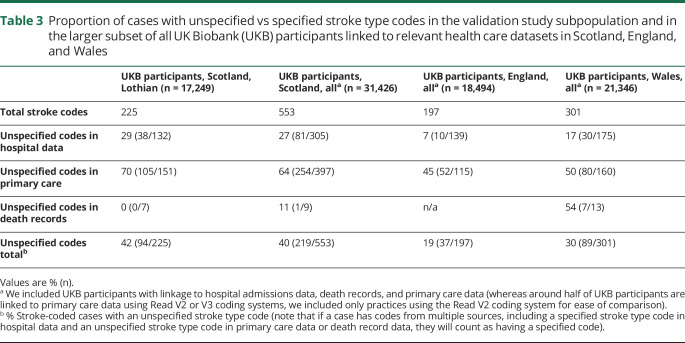
Proportion of cases with unspecified vs specified stroke type codes in the validation study subpopulation and in the larger subset of all UK Biobank (UKB) participants linked to relevant health care datasets in Scotland, England, and Wales

### Interadjudicator agreement

All vignettes were independently reviewed by 2 adjudicators. For assigning a diagnosis of stroke vs not, interadjudicator agreement was 91% and Cohen kappa very good at 0.7 (95% CI, 0.6–0.8); for stroke/TIA vs not, agreement was 93% and Cohen kappa very good at 0.7 (95% CI, 0.6–0.9). Agreement for assigning a stroke type (ischemic stroke vs ICH vs SAH vs uncertain stroke type) was 99% and Cohen kappa excellent at 0.98 (95% CI, 0.9–1).

### Code accuracy

**Figure 2:**
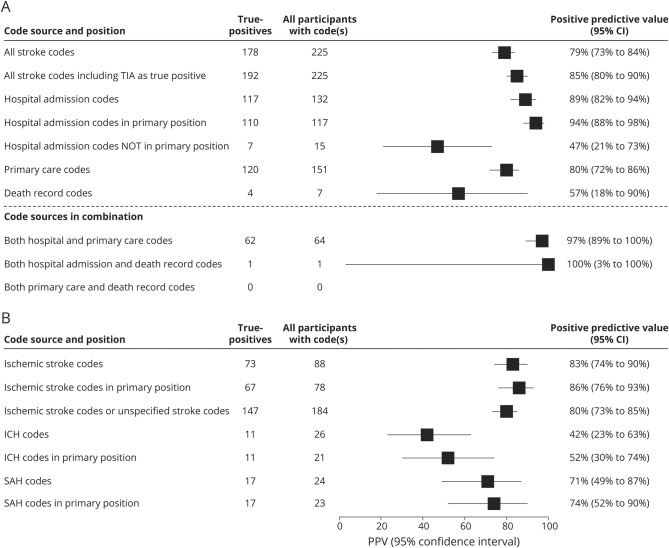
Positive predictive values (PPVs) of stroke codes PPVs of stroke codes stratified by code source (A) and code type (B). Primary position: includes primary care codes, where no code position is specified, and only primary position hospital admission codes. CI = confidence interval; ICH = intracerebral hemorrhage; SAH = subarachnoid hemorrhage.

The overall PPV of case ascertainment for all 225 stroke-coded cases from all sources combined was 79% (95% CI, 73%–84%). When broken down by code source, PPV was highest for the 132 cases with hospital admission codes (89%; 95% CI, 82%–94%), and higher still when limiting analyses to primary position hospital codes only (94%; 95% CI, 88%–98%), at the expense of losing a few (<10%) true-positive cases. PPV for the 151 cases with primary care codes was 80% (95% CI, 72%–86%). Only 7 cases had death record codes, with PPV 57% (95% CI, 18%–90%), the wide CIs indicating limited precision of this estimate. For cases with both a hospital admission and a primary care code, PPV was very high (97%; 95% CI, 89%–100%), but only a third of the cases fell into this category (figure 2A).

As regards the accuracy of identifying stroke pathologic type (for these estimates, a case would be counted in ≥1 analysis if it had ≥1 unique stroke type code), PPV among the 88 participants with an ischemic stroke code was 83% (95% CI, 74%–90%); restricting to primary position codes (for those cases from hospital admission data) increased this to 86% (95% CI, 76%–93%), with loss of <10% of true-positive cases (figure 2B). Given that ischemic stroke is the most common pathologic stroke type, and hence an unspecified stroke code is much more likely to signify an ischemic rather than a hemorrhagic stroke case, we calculated the PPV for ischemic stroke of the 184 ischemic and unspecified stroke codes combined. While this resulted in a slightly lower PPV of 80% (95% CI, 73%–85%), it approximately doubled the number of true-positive ischemic stroke cases identified. The proportion of true-positive ischemic stroke cases among all cases with an unspecified code was 77%. Accuracy of hemorrhagic stroke codes was lower but the small numbers of coded cases resulted in limited precision: PPV among 26 participants with an ICH code was 42% (95% CI, 23%–63%) and among 24 participants with a SAH code was 71% (95% CI, 49%–87%). Restricting to primary position codes increased the PPV of both ICH and SAH codes without losing any true-positive cases.

**Figure 3:**
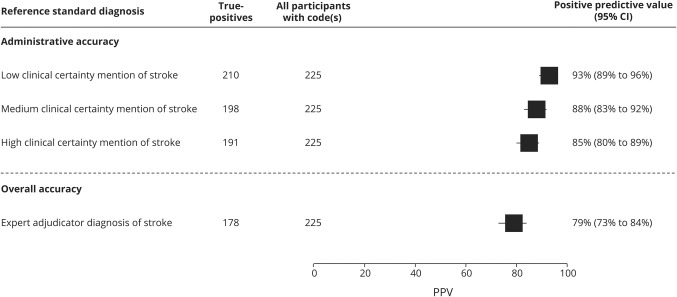
Assessing administrative vs overall accuracy High clinical certainty mentions of stroke: “stroke,” “probable stroke,” “presumptive stroke,” “consistent with stroke,” “compatible with stroke,” “likely stroke,” “treated as stroke” or equivalent stroke terms (ICH, SAH, intracerebral hemorrhage, subarachnoid hemorrhage, ischemic stroke, infarct). Medium clinical certainty mentions of stroke: including above plus “possible stroke,” “suspected stroke,” “impression of stroke,” “suggestive of stroke,” “query stroke,” or equivalent stroke terms (ICH, SAH, intracerebral hemorrhage, subarachnoid hemorrhage, ischemic stroke, infarct). Low clinical certainty mentions of stroke include above plus “TIA” and “transient ischaemic attack” with any level of certainty preceding it. The hierarchy of clinical certainty was based on the ICD-10 clinical coding instruction manual (isdscotland.org/Products-and-Services/Terminology-Services/Clinical-Coding- Guidelines/, April 2010 version), which is used by the coding departments in UK hospitals. CI = confidence interval; PPV = positive predictive value.

PPV when measuring only administrative accuracy was 93%, decreasing to 79% when measuring overall accuracy. This demonstrates the importance of expert-led adjudication in validation studies, as ignoring it would lead to falsely inflated PPVs (figure 3).

### Further exploration of false-positive codes

The 47 false-positive stroke-coded cases had a similar proportion of unspecified codes and median age (at date of recruitment or first stroke code) as true-positives. False-positive cases were more likely to have a primary care code and to have died by the end of follow-up, and were slightly more likely to be female, to have a hemorrhagic stroke code, and to have an earlier first stroke code date (table 1).

Alternative diagnoses for the 47 false-positives included TIA (14/47, 30%); secondary intracranial bleed not due to SAH or ICH (e.g., traumatic, tumor-related, bleeding into a cerebral infarct) (11/47 [23%]); radiologic finding of a suspected old vascular lesion (usually incidental) (8/47 [17%]); and possible or probable alternative diagnosis (e.g., migraine, demyelination, seizure, or another diagnosis) (14/47 [30%]).

Among the 15 participants with a false-positive ICH code, 7/15 had another primary stroke type ± secondary ICH, 4/15 intratumor bleed, 3/15 traumatic or other intracranial bleed, and 1/15 no obvious reason for an ICH code. Among the 7 participants with a false-positive SAH code, 4/7 were traumatic intracranial bleed, 2/7 another primary stroke type ± secondary SAH component, and 1/7 asymptomatic aneurysm.

**Figure 4:**
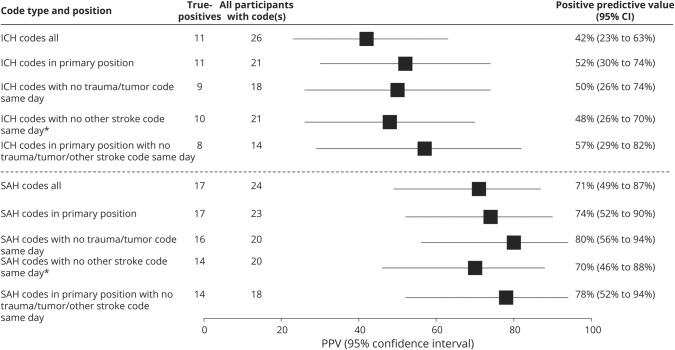
Exploratory analyses to improve accuracy of hemorrhagic stroke codes *Excluding other stroke code same day: excluded cases with a diagnostic code for >1 stroke pathologic type on the same day. This was done because a patient who has one pathologic stroke type (e.g., ischemic stroke) can sometimes develop a complication and subsequent brain scan appearances similar to another pathologic stroke type (e.g., a patient with ischemic stroke can have a bleed in the brain as a result of the ischemic stroke, which could lead to a false diagnosis of an intracerebral hemorrhage [ICH]). CI = confidence interval; PPV = positive predictive value; SAH = subarachnoid hemorrhage.

Because performance for hemorrhagic stroke codes was suboptimal, having observed that the more common alternative diagnoses were traumatic and intratumor bleeds or stroke complicated by a bleed, we performed additional analyses to explore the effects of different code inclusion criteria on numbers of cases, numbers of true-positives, and PPV for ICH and SAH. For both ICH and SAH, we could increase PPV but at the expense of failing to detect some true-positive cases. However, numbers of cases were too small to draw firm conclusions (figure 4).

### Stroke type and subtype distributions

With information from the EPR, a stroke specialist was able to assign a pathologic stroke type to 177 of the 178 true-positive stroke cases: 149 (84%) were ischemic, 11 (6%) ICH, and 17 (10%) SAH. Depending on the subclassification system used, an ischemic subtype could be determined for 37%–87% of cases, an ICH subtype for 27%–100% of cases, and SAH subtype for 94% of cases (figure e-3, dryad.org/10.5061/dryad.w9ghx3fk0).

## Discussion

Our results suggest that stroke cases, and cases of ischemic stroke type, can be ascertained in UKB through linked coded data with sufficient accuracy for use in many genetic and epidemiologic research studies without further expert validation, since PPVs were generally at least 80% despite stringent adjudication criteria. Primary care is an important code source with ≥1/3 of the cases ascertained exclusively via primary care data in this UK setting. Code accuracy appeared slightly better for hospital admission compared to primary care codes. There were insufficient data to draw firm conclusions regarding the accuracy of death record or hemorrhagic stroke type codes. Including only the primary position codes from hospital admission data increased PPV without loss of a substantial proportion of true-positive cases. In the subpopulation of Scotland studied, only around 60% of codes were specific for a stroke type; however, using all available relevant medical information from the EPR, an expert adjudicator could assign a stroke type to 99%, and a more detailed stroke subtype to between a third and almost 90% of cases, depending on the subclassification system used. A higher proportion of participants had a specified stroke type code in the English and Welsh data, but the accuracy of these codes needs further investigation. We also demonstrated that, at least in this setting, considering only the administrative accuracy of stroke diagnosis codes (rather than overall accuracy based on an expert review of the full EPR) may give falsely inflated PPVs.

Acceptable levels of accuracy, and the relative importance of different accuracy metrics, depend on the context.^8^ UKB is primarily used for research into the genetic and nongenetic determinants of disease.^3^ In such analyses, it is important to ensure that a high proportion of participants identified as disease cases truly do have the disease (high PPV) to minimize bias in effect estimates, while aiming to optimize statistical power by ascertaining as many true-positive cases as possible (optimizing sensitivity, but not necessarily maximizing it, as this may compromise the PPV). A high specificity (the proportion of participants without the disease that do not receive a stroke code) is crucial in obtaining a high PPV, but is not sufficient in and of itself. In population-based prospective cohorts where the proportion of all participants who are true-positives for the disease (in this case stroke) is generally low, the proportion of all participants incorrectly classified as having stroke (false-positives) will generally be low (giving high specificity), even if the absolute number of false-positives is high compared to the absolute number of true-positives (low PPV).^8^ Providing appropriate codes are used, both the specificity and negative predictive value of routinely collected health care data to identify disease cases in population-based studies are usually very high (96%–100%).^4^ For these reasons, we focused on estimating the PPV of using routinely collected health care data to identify stroke cases in UKB, and on assessing the effects of different code and source selections on both PPV and number of true-positive cases.

Strengths of this study include (1) the overall large number of participants; (2) inclusion of primary care and death record codes for which accuracy data have previously been limited; (3) creation of vignettes using preset criteria from the EPR for 97% of otherwise eligible stroke-coded cases, thus avoiding the bias of selecting participants with a higher prior probability of having had a stroke; (4) blinding of adjudicators to codes and each other's diagnoses; (5) assessment of interadjudicator reliability; (6) further analysis of false-positive codes; and (7) assessment of both administrative and overall accuracy, demonstrating the importance of an expert-led reference standard in validation studies.

There are some limitations: (1) small numbers precluded robust conclusions about the accuracy of death record and hemorrhagic stroke codes; (2) our lack of access to the full free text EPRs held exclusively by the primary care system may have led to slightly more conservative PPV estimates (although we would not expect this to be a major issue for stroke), as almost all stroke cases have acute inpatient or outpatient management in secondary care (in keeping with this is that we were able to find secondary care records for 97% of the cases) and information provided from primary to secondary care was available in the hospital EPR; (3) the potential difficulty in differentiating between definite false-positive and uncertain cases from retrospective review of medical records, which may have further reduced the estimated PPVs; and (4) the restriction of the current study to a Scottish subpopulation of the UKB participants (although we have no reason to suspect substantial variation in the results considering that the code generation process across the United Kingdom follows similar pathways and given the broadly similar distribution of code sources in the wider UKB population across England, Scotland, and Wales).

The present study is limited to validating the accuracy of codes for symptomatic stroke (as defined by the WHO). Subclinical cerebrovascular disease (such as that detected by brain imaging), which is more common, particularly in older people,^9^ would not be readily ascertained by the linked, coded health care data sources used to follow the health of UKB participants, but can be detected through brain imaging conducted as part of the UKB multimodal imaging study of 100,000 of its participants.

These results are largely consistent with those from our earlier systematic review,^4^ where PPVs for stroke-specific ICD-10 hospital admission codes ranged from 79% to 83%,^10,11^ and for ischemic stroke from 86% to 88%.^10,12,13^ Our results for ICH and SAH code accuracy among a small number of participants were worse than those from the 2 previous United Kingdom–based studies of ICD-10 or Read codes.^13,14^ This may be in part because these studies checked only (or mainly) for administrative accuracy, hence inflating the PPVs. Our results were similar to a Danish study, which also used an expert review of medical records to check for overall accuracy.^10^ To our knowledge, there are no previous validation studies of stroke-specific UK primary care Read codes or death record codes for diagnosis of all types of stroke, nor of the effect of combining primary care and hospital admission coded data.

Further work will be required to further explore the accuracy of hemorrhagic stroke and death record codes in larger numbers of cases by expanding this work to other UKB recruitment locations, which will also enable assessment of the generalizability of these results to other regions of the United Kingdom. In our Scottish subpopulation, while over one-third of the codes were not specific for a stroke type, expert adjudication allowed a stroke type to be assigned for almost all cases. Future studies should investigate ways of automating this process, as well as methods for determining more detailed stroke subtypes.

Our results suggest that stroke and ischemic stroke cases in UKB can be identified through linked coded data with sufficient accuracy for many genetic and epidemiologic studies, while there are currently insufficient data to draw definite conclusions about the accuracy of hemorrhagic stroke codes.

## Glossary

CI: confidence interval
EPR: electronic patient record
ICD: International Classification of Diseases
ICH: intracerebral hemorrhage
PPV: positive predictive value
SAH: subarachnoid hemorrhage
UKB: UK Biobank
